# Role of cardiovascular magnetic resonance imaging and cardiopulmonary exercise test in predicting composite clinical outcomes in patients with hypertrophic cardiomyopathy

**DOI:** 10.1371/journal.pone.0285887

**Published:** 2023-05-16

**Authors:** Ji-won Hwang, Sang-Chol Lee, Darae Kim, Jihoon Kim, Eun Kyoung Kim, Sung-A. Chang, Sung-Ji Park, Sung Mok Kim, Yeon Hyeon Choe, Seung Woo Park

**Affiliations:** 1 Division of Cardiology, Department of Medicine, Ilsan Paik Hospital, Inje University School of Medicine, Goyang, Korea; 2 Division of Cardiology, Department of Medicine, Samsung Medical Center, Sungkyunkwan University School of Medicine, Seoul, Korea; 3 Cardiovascular Imaging Center, Heart Vascular Stroke Institute, Samsung Medical Center, Sungkyunkwan University School of Medicine, Seoul, Korea; 4 Department of Radiology, Samsung Medical Center, Sungkyunkwan University School of Medicine, Seoul, Korea; Ohio State University, UNITED STATES

## Abstract

We aimed to evaluate the additive value of cardiovascular magnetic resonance imaging (CMR) and cardiopulmonary exercise test (CPET) to predict clinical outcomes in patients with HCM. We enrolled 373 patients with HCM and normal left ventricular systolic function who underwent CPET and CMR. The primary outcome was a clinical composite of all-cause death, cardiac transplantation, stroke, heart failure requiring hospitalization and defibrillator implantation. During a follow-up of 70.70 ± 30.74 months, there were 84 composite clinical events. Peak oxygen consumption during CPET was significantly lower (18.51±13.25 vs. 24.59±13.28 mL/kg/min, p < 0.001) and abnormal hemodynamic response to exercise was more frequently detected (41.7 vs. 20.8%, p<0.001) in the group with composite clinical events. The extent of late gadolinium enhancement was larger in the event group (15.39±10.53 vs. 11.97±9.53%LV, p<0.001). Selective parameters were added stepwise to conventional clinical parameters; the final model, where CPET and CMR parameters were added, was verified to have the highest increment value for clinical outcome prediction (p<0.001). This study demonstrated that CPET and CMR findings may be important clinical tools for risk stratification in HCM. Exercise capacity was an independent predictor of composite outcomes in patients with HCM, with incremental value as a risk factor when added to the alleged parameters. These findings could help physicians monitor and manage patients with HCM in the real clinical field.

## Introduction

Hypertrophic cardiomyopathy (HCM) is a cardiac disease characterized by inappropriate myocardial hypertrophy and a non-dilated left ventricle with frequent myocardial fibrosis. It is associated with variable clinical expressions and outcomes [1, 2].

Reduced exercise capacity is common in patients with HCM, affecting these patients over a broad spectrum of clinical severity [3]. Exercise stress testing has been shown to be useful for the assessment of functional capacity and risk stratification, evaluation of symptoms, and monitoring the response to therapy in patients with HCM [4]. Peak oxygen consumption (peak VO_2_) during cardiopulmonary exercise testing (CPET) is known to be a useful marker for the functional capacity of patients with HCM [5, 6].

Meanwhile, myocardial fibrosis in HCM has been reported to be responsible for poor clinical outcomes [7–9]. It is most commonly detected and quantified with cardiac magnetic resonance imaging (CMR) using late gadolinium enhancement (LGE) [10]. Previous reports have shown that the presence of LGE has an independent value as a prognostic factor in HCM. It has also been proposed as a factor to be considered when an implantable cardioverter-defibrillator is considered for primary prevention of sudden cardiac death [10–12].

Moreover, CPET and CMR have been described as being able to further aid in risk stratification for future clinical events [13–17]. Apart from conventional factors, additional risk stratification could be beneficial and useful for better prediction of clinical outcomes.

We sought to assess whether the extent of myocardial fibrosis depicted on CMR and exercise capacity estimated by CPET could help in determining the outcomes, assessed as independent risk factors or as additive parameters to conventional clinical parameters, in patients with HCM.

## Methods

### Study population and clinical characteristics

The study was performed using data obtained from the prospective observational HCM registry of the Samsung Heart Vascular Stroke Institute, Seoul, Korea. A total of 591 prospectively enrolled patients, who were diagnosed with HCM and underwent CMR between 2008 and 2015, were included. The echocardiographic criteria for inclusion in the HCM registry included the following definition of guidelines: HCM is defined by the presence of increased left ventricular (LV) wall thickness that is not solely explained by abnormal loading conditions [18, 19]. Patients with uncontrolled hypertension, uncontrolled ventricular arrhythmias, severe valvular diseases, other concomitant systemic diseases–including malignancy–and poor echocardiographic windows for analysis were excluded [3, 20–22].

The study population comprised 373 patients who were able to perform a cardiopulmonary exercise test (CPET) using a treadmill and presented with normal LV systolic function (ejection fraction ≥ 50%). CPET and CMR were performed according to a standard protocol at baseline.

All clinical characteristics were obtained by reviewing medical records. The presenting symptoms, such as dyspnea (New York Heart Association [NYHA] functional classification) or chest pain, were recorded, and a medical history of syncope or sudden cardiac death was obtained. History of other medical conditions, such as the presence of hypertension, diabetes mellitus, or atrial fibrillation, was also acquired. Information on family history (defined as history in first-degree relatives) of sudden cardiac death or HCM was also obtained. A 24-h electrocardiogram (ECG) Holter monitoring was also performed in the study population for risk stratification.

This study was approved by the Institutional Review Board of Samsung Medical Center, and the requirement for informed consent was waived.

### Echocardiographic study and CPET

Conventional two-dimensional echocardiography was performed and echocardiographic parameters were obtained according to the guidelines. LV end-diastolic and end-systolic volumes were measured from apical two- and four-chamber views. The ejection fraction was calculated using Simpson’s rule. LV mass was calculated using the formula proposed by Devereux et al. and was corrected by body surface area to derive the LV mass index (LVMI) [23]. The left ventricular outflow tract (LVOT) was scanned using continuous Doppler to measure the maximal velocity and estimated pressure gradient in apical three- or five-chamber views. Left ventricular outflow tract obstruction (LVOTO) was defined as a pressure gradient of ≥ 30 mmHg at rest [19, 22, 24–26].

Left atrial volume was measured using the biplane-modified Simpson’s method and adjusted to the body surface area (left atrium volume index, LAVI). Early diastolic mitral annular velocity at the septal side (e’) and peak early diastolic transmitral flow velocity (E) were measured. E/e’ was calculated from the aforementioned values [27].

CPET was performed with symptom-limited treadmill exercise along with respiratory gas exchange analysis in all patients using the Bruce protocol [28–30]. Twelve-lead electrocardiography was performed with the use of conventional chest lead positioning before exercise, at each stage, and after stress test. Blood pressure was recorded every 2 min. Oxygen consumption (peak VO_2_) was measured using a Medical Graphics metabolic cart (St. Paul, MN, USA). The highest peak VO_2_ over a period of exercise time was expressed as absolute peak VO_2_ or normalized peak VO_2_ (percentage of age, sex, and weight predicted). Exercise was terminated if there was marked dyspnea, fatigue, chest discomfort, > 2-mm ST depression, or after 15 min of the protocol (15.6 metabolic equivalents, MET). In addition, abnormal hemodynamic response to exercise was defined as follows: (1) blunted blood pressure (BP) response, < 20 mmHg increase in systolic BP, or (2) hypotensive response, > 20 mmHg decrease in systolic BP from baseline BP, or an initial rise in systolic BP, followed by a decrease of > 20 mmHg in systolic BP [31, 32].

### CMR imaging acquisition and analysis

CMR was performed using a 1.5 T system (Magnetom Avanto, Syngo MR B15 version; Siemens Medical Solutions, Erlangen, Germany) with a 32-channel phased-array receiver coil with repeated breath-holding. The CMR protocol consisted of cine, T2-weighted (T2W), first-pass perfusion, and LGE imaging. Cine imaging was performed with balanced steady-state free precession sequences along the long and short axes from the apex to the base of the LV. After localization, steady-state free precession cine images were acquired with contiguous short-axis slices (slice thickness, 6 mm; gap, 4 mm) to quantify LV function and volume.

Standard LGE images were acquired after intravenous administration of gadobutrol (0.15 mmol/kg Gadovist; Bayer Healthcare, Berlin, Germany), followed by a flush of 20 mL saline at the same rate. The LGE images from long -, short -, and apical 4-chamber views were acquired using a multi-shot turbo field echo breath-hold sequence with a phase-sensitive inversion recovery method. The inversion time was usually 280–360 ms. The LGE images were evaluated 10 min after gadolinium administration using a multi-shot turbo field echo breath-hold sequence. The field of view and image matrix were 35 × 35 cm and 256 × 256 cm [33]. LGE was considered present when the signal intensity of the index myocardial segment was 6 SD greater than the remote normal myocardial signal. LGE volume was calculated by summing the areas of LGE in all short-axis slices and was expressed as the volumetric proportion of the total LV myocardium (%LV). The CMR scans were visually interpreted by two experienced readers who were blinded to the clinical and laboratory data. The cutoff value of extensive LGE was defined as 15%LV, in accordance with previous studies that showed that this proportion of LGE by quantitative contrast enhanced CMR could help in assessing event risk [34].

### Clinical outcomes

Clinical outcomes were evaluated through medical record reviews and regular telephone interviews. The primary outcome was a clinical composite of all-cause death, cardiac transplantation, stroke, heart failure requiring hospitalization (including new-onset atrial fibrillation), and implantable cardiac defibrillator implantation [24, 26]. In addition, secondary outcome analyses were performed against each component of the composite clinical outcome.

### Statistical analysis

Continuous variables were compared using Student’s t-test or the Wilcoxon rank-sum test, where applicable, and are presented as mean ± standard deviation or median with interquartile range (IQR). Categorical data were tested using Fisher’s exact test or chi-square test, as appropriate. Cox proportional analysis was used to calculate the hazard ratio (HR) and 95% confidence interval (CI) to compare the risk of composite clinical outcomes. A multivariable Cox proportional hazards regression model, adjusted for age and sex, was used to identify independent predictors of composite clinical outcomes. Covariates that were either statistically significant during univariate analysis or clinically relevant were included in the multivariable models. We compared several receiver operating characteristic (ROC) curves using the area under the curve (AUC) comparison analysis method.

The variables selected for the multivariable model analyses were variables that were found to be either significant in univariable analyses (p < 0.2) or represented to be clinically meaningful parameters [35]. In addition to clinical risk factors, the incremental prognostic value of additive parameters was assessed using the net reclassification improvement (NRI) and integrated discrimination improvement (IDI) index. MODEL 1 was established for combined conventional parameters, including age, sex, history of syncope, and non-sustained ventricular tachycardia (VT) on Holter monitoring [36].

All tests were two-tailed, and statistical significance was set at p < 0.05. All analyses were performed using SPSS software (version 23.0; SPSS Inc., Chicago, IL, USA) and R statistical software version 3.6.1 (R Foundation for Statistical Computing, Vienna, Austria).

## Results

### Baseline characteristics and composite clinical outcomes

Baseline clinical characteristics of the patients are shown in Table 1. A total of 373 patients were referred for exercise stress testing and CMR. Patients were divided into two groups according to the presence or absence of composite clinical outcomes, comprising 84 and 289 patients, respectively. The patients in the group with composite clinical outcomes were older (56.08 ± 10.23 years vs. 52.16 ± 11.22 years, p = 0.003). The sex ratio (male) of the patients was not significantly different between the two groups (81.0% vs. 84.4% males, p = 0.51).

**Table 1:** Baseline clinical characteristics of the study population.

Clinical parameters	Composite clinical outcomes (-) (N = 289)	Composite clinical outcomes (+) (N = 84)	p-value
age, years	52.16 ± 11.22	56.08 ± 10.23	0.004
Sex ratio (Male)	244 (84.4%)	68 (81.0%)	0.50
family history of HCMP	19 (6.6%)	11 (13.1%)	0.07
family history of sudden cardiac death	35 (12.1%)	11 (13.1%)	0.85
history of syncope	19 (6.6%)	13 (15.5%)	0.015
history of chest pain	51 (17.6%)	13 (15.5%)	0.74
past medical history			
atrial fibrillation	8 (2.8%)	11 (13.1%)	0.001
hypertension	119 (41.2%)	38 (45.2%)	0.53
diabetes mellitus	31 (10.7%)	16 (19.0%)	0.06
NYHA class ≥ II	42 (14.5%)	36 (42.9%)	< 0.001
History of non-sustained VT on holter	22 (7.6%)	24 (28.6%)	< 0.001
NT-proBNP (pg/mL)	536.58 ± 890.96	897.29 ± 905.61	0.002

Data are presented are number of patients (percent) or average ± standard deviation.

HCM; hypertrophic cardiomyopathy, VT; ventricular tachycardia

The median follow-up period was 74.7 months (interquartile range, 50.0–103.3 months). Nine patients died regardless of cause (10.7%), and only three patients experienced cardiac-related events. Cardiac transplantation, stroke, heart failure requiring hospitalization, and implantable cardiac defibrillator implantation occurred in 1 (1.2%), 24 (28.6%), 55 (65.5%), and 14 (16.7%) patients, respectively. Among the composite clinical outcomes, heart failure that required hospitalization had the highest occurrence rate in this registry.

### Association of clinical parameters and events

The composite of clinical events was associated with dyspnea on exertion (NYHA functional class II, 42.9% vs. 14.5%, p < 0.001). Patients with clinical events presented with a higher proportion of history of syncope (15.5% vs. 6.6%, p = 0.015). A history of atrial fibrillation (AF) (13.1% vs. 2.8%, p = 0.008) and non-sustained VT on 24-hour ECG monitoring (28.6% vs. 7.6%, p < 0.001) were also more frequently detected in patients with clinical events.

### Echocardiographic, CMR, and CPET parameters

The parameters obtained through echocardiography, CMR, and CPET are listed in Table 2. On echocardiography, maximal LV wall thickness (18.67 ± 4.60 mm vs. 17.31 ± 4.07 mm, p = 0.009) and LV end-diastolic dimension (LVEDD) (47.37 ± 5.28 mm vs. 48.70 ± 4.88 mm, p = 0.031) were significantly higher in patients with clinical events. The incidence of LVOTO was higher in the event group (28.6% vs. 14.9%, p = 0.006). The composite outcome was also associated with larger LAVI (52.18 ± 19.22 mL/m^2^ vs. 40.73 ± 15.62 mL/m^2^, p < 0.001) and higher E/e’ (14.07 ± 5.70 vs. 11.39 ± 4.29, p < 0.001).

**Table 2:** The parameters of echocardiography, exercise stress test, and cardiac magnetic resonance on study population.

	Composite clinical outcomes (-) (N = 289)	Composite clinical outcomes (+) (N = 84)	p-value
baseline echocardiographic parameters			
Maximal left ventricular wall thickness (mm)	17.31 ± 4.07	18.67 ± 4.60	0.009
Presence of left ventricular outflow tract obstruction	43 (14.9%)	24 (28.6%)	0.006
Left ventricular ejection fraction (%)	65.93 ± 6.06	64.66 ± 7.32	0.15
Left ventricle end-diastolic dimension (mm)	48.70 ± 4.88	47.37 ± 5.28	0.031
Left ventricle end-systolic dimension (mm)	28.49 ± 3.73	28.11 ± 4.45	0.42
Interventricular septum (mm)	13.95 ± 5.06	15.60 ± 5.35	0.010
Left ventricular posterior wall (mm)	9.77 ± 1.71	9.96 ± 2.14	0.39
Relative wall thickness	0.40 ± 0.08	0.43 ± 0.14	0.14
Left atrium size (mm)	42.05 ± 6.14	45.18 ± 7.63	0.001
Left atrium volume index (mL/m^2^)	40.73 ± 15.62	52.18 ± 19.22	< 0.001
E velocity (m/s)	0.62 ± 0.18	0.65 ± 0.21	0.23
e’ velocity (cm/s)	5.79 ± 1.85	5.08 ± 1.96	0.002
E/e’ ratio	11.39 ± 4.29	14.07 ± 5.70	< 0.001
cardiopulmonary exercise test parameters			
exercise duration (sec)	585.90 ± 137.79	502.48 ± 145.89	< 0.001
exercise duration (< 475 sec)	54 (18.7%)	38 (45.2%)	< 0.001
exercise duration (≥ 475 sec)	235 (81.3%)	46 (54.8%)	< 0.001
peak systolic blood pressure	168.19 ± 37.61	152.86 ± 45.22	0.005
peak heart rate	143.53 ± 28.30	133.93 ± 30.23	0.007
abnormal response of blood pressure	60 (20.8%)	35 (41.7%)	< 0.001
Exercise capacity (Peak VO_2_) (mL/Kg/min)	24.59 ± 13.28	18.51 ± 13.25	< 0.001
Lower peak VO_2_ (≤27.4 mL/Kg/min)	125 (43.3%)	61 (72.6%)	< 0.001
baseline CMR parameters			
LGE (%LV)	11.97 ± 9.53	15.39 ± 10.53	0.005
The presence of LGE (>15%LV)	82 (28.4%)	42 (50.0%)	< 0.001
Left ventricular ejection fraction (%)	70.01 ± 6.76	66.39 ± 10.03	0.002
Left ventricle end-diastolic volume (mL)	146.04 ± 114.32	133.54 ± 26.25	0.32
Left ventricle end-systolic volume (mL)	41.65 ± 16.34	44.67 ± 15.88	0.13
Stroke volume (mL)	96.27 ± 19.71	88.85 ± 23.37	0.004
Cardiac output (L)	7.22 ± 10.50	5.89 ± 1.44	0.25
Left ventricular mass index (g/m2)	89.56 ± 27.86	95.63 ± 31.01	0.09

Data are presented are number of patients (percent) or average ± standard deviation.

LGE; Late gadolinium enhancement

CMR imaging revealed that the extent of LGE was larger in the event group (15.39 ± 10.53%LV vs. 11.97± 9.53%LV, p = 0.005). An ROC curve analysis was performed to obtain the cutoff value of the amount of LGE associated with prognosis. The cutoff value was 15.29% on the ROC curve, which was consistent with the findings of previous studies that defined a significant amount of LGE that could predict worse prognosis [34]. The proportion of patients with extensive LGE (≥ 15%LV) according to our cutoff value criteria was also significantly higher in the event group (50.0% vs. 28.4%, p < 0.001).

On exercise stress testing, the group with composite clinical outcomes showed lower peak VO_2_ (18.51 ± 13.25 mL/kg/min vs. 24.59 ± 13.28 mL/kg/min, p < 0.001) and shorter exercise duration (p < 0.001). Furthermore, an abnormal hemodynamic response to exercise (41.7% vs. 20.8%, p < 0.001) was more common in the event group. An ROC curve analysis was performed to obtain the cutoff value of the amount of peak VO_2_ associated with prognosis, as the cutoff value was confirmed to be 27.4 (mL/Kg/min).

### Multivariate analysis of variables associated with composite clinical outcomes

The Cox regression analysis of the variables evaluated to predict composite clinical outcomes is shown in Table 3. Multivariate Cox proportional analysis, adjusted for age and sex, revealed that non-sustained VT on Holter (HR [95% CI]: 2.460 [1.444–4.190], p = 0.001), E/e’ ratio (1.051 [1.006–1.098], p = 0.027), abnormal hemodynamic response to exercise (1.661 [4.025–2.692], p = 0.04), and lower exercise capacity (≤ 27.4 mL/kg/min) (1.724 [1.004–2.962], p = 0.048) were significantly correlated with composite outcome (Table 3).

**Table 3:** Cox-proportional analysis as adjusted with parameters for the prediction of composite clinical outcomes and single components.

						univariable							multivariable		
				HR		95% CI		p-value		beta		HR	95% CI		p-value
Clinical data															
Age, years				1.034		1.012–1.057		0.002		0.034		1.034	1.010–1.059		0.005 *
Sex (Male)				1.198		0.694–2.067		0.52		-0.512		0.599	0.324–1.107		0.102 *
History of syncope				2.339		1.293–4.233		0.005		0.402		1.494	0.792–2.820		0.22 *
Atrial fibrillation				3.606		1.909–6.810		< 0.001		0.216		1.242	0.537–2.870		0.61
History of non-sustained VT on holter				3.623		2.250–5.833		< 0.001		0.900		2.460	1.444–4.190		0.001 *
Baseline echocardiographic parameters															
Maximal left ventricular wall thickness (mm)				1.065		1.019–1.115		0.006		0.005		1.005	0.955–1.057		0.86
Presence of LVOT obstruction				1.973		1.227–3.173		0.005		0.224		1.251	0.679–2.303		0.47
Left ventricle end-diastolic dimension (LVEDD) (mm)				0.956		0.917–0.997		0.035		-0.039		0.961	0.915–1.010		0.12 *
Left atrium volume index (LAVI) (mL/m^2^)				1.018		1.012–1.024		< 0.001		0.009		1.009	0.997–1.020		0.13 *
E/e’ ratio				1.101		1.062–1.142		< 0.001		0.050		1.051	1.006–1.098		0.027 *
Cardiopulmonary exercise test parameters															
Abnormal hemodynamic response to exercise				2.609		1.686–4.039		< 0.001		0.507		1.661	1.024–2.692		0.040 *
Exercise capacity (peak VO_2_ ≤27.4 mL/Kg/min)				3.271		2.008–5.328		< 0.001		0.545		1.724	1.004–2.962		0.048 *
Baseline CMR parameters															
The presence of LGE (>15%LV)				2.410		1.564–3.713		< 0.001		0.381		1.464	0.876–2.445		0.15 *
Left ventricular ejection fraction (%)				0.953		0.930–0.976		< 0.001		-0.022		0.979	0.952–1.006		0.12 *
				All cause death						Cardiac transplantation					
				Adjusted HR (95% CI)					p-value	Adjusted HR (95% CI)				p-value	
Clinical data															
Age, years				1.158 (0.018–1.318)					0.026	1.00 (0.816–1.226)				NA	
Sex (Male)				3.904 (0.353–43.224)					0.27	1.00 (0.003–323.311)				NA	
History of syncope				0.245 (0.015–4.080)					0.33	1.00 (0.002–571.492)				NA	
Atrial fibrillation				9.580 (0.847–108.325)					0.07	1.00 (0–16640.166)				NA	
History of non-sustained VT on holter				0.531 (0.066–4.255)					0.55	1.00 (0.002–623.535)				NA	
Baseline echocardiographic parameters															
Maximal left ventricular wall thickness (mm)				0.975 (0.814–1.166)					0.78	1.00 (0.551–1.814)				NA	
Presence of LVOT obstruction				25.160 (3.154–200.705)					0.002	1.00 (0.004–236.130)				NA	
Left ventricle end-diastolic dimension (LVEDD) (mm)				1.110 (0.952–1.295)					0.18	1.00 (0.608–1.643)				NA	
Left atrium volume index (LAVI) (mL/m^2^)				0.960 (0.906–1.017)					0.16	1.00 (0.814–1.228)				NA	
E/e’ ratio				0.934 (0.787–1.110)					0.44	1.00 (0.562–1.778)				NA	
Cardiopulmonary exercise test parameters															
Abnormal hemodynamic response to exercise				10.568 (1.451–76.975)					0.020	1.00 (0.005–205.019)				NA	
Exercise capacity (peak VO_2_ ≤27.4 mL/Kg/min)				0.353 (0.033–3.753)					0.39	1.00 (0.012–80.763)				NA	
Baseline CMR parameters															
The presence of LGE (>15%LV)				27.120 (3.024–243.227)					0.003	1.00 (0.006–160.635)				NA	
Left ventricular ejection fraction (%)				0.992 (0.900–1.092)					0.87	1.00 (0.796–1.257)				NA	
Stroke						Heart failure hospitalization						Defibrillator implantation			
Adjusted HR (95% CI)				p-value		Adjusted HR (95% CI)				p-value		Adjusted HR (95% CI)		p-value	
1.052 (1.003–1.103)				0.038		1.031 (1.001–1.062)				0.040		1.007 (0.953–1.064)		0.81	
1.313 (0.424–4.062)				0.64		0.680 (0.327–1.413)				0.30		0.131 (0.012–1.423)		0.10	
NA				0.98		2.431 (1.174–5.030)				0.017		3.203 (0.920–11.151)		0.07	
6.846 (1.650–28.401)				0.008		1.301 (0.448–3.781)				0.63		0.065 (0.003–1.222)		0.07	
1.587 (0.547–4.602)				0.40		2.111 (1.075–4.143)				0.030		3.476 (0.876–13.796)		0.08	
1.021 (0.921–1.132)				0.69		1.019 (0.958–1.084)				0.54		1.142 (1.022–1.277)		0.019	
0.820 (0.237–2.842)				0.75		0.953 (0.436–2.085)				0.90		3.051 (0.409–22.786)		0.28	
0.966 (0.879–1.061)				0.47		0.941 (0.883–1.001)				0.054		0.962 (0.843–1.098)		0.57	
0.997 (0.975–1.019)				0.77		1.015 (1.001–1.029)				0.034		1.041 (1.008–1.075)		0.015	
0.994 (0.899–1.100)				0.91		1.078 (1.021–1.138)				0.007		0.879 (0.734–1.053)		0.16	
1.830 (0.750–4.466)				0.18		1.161 (0.632–2.132)				0.63		4.012 (1.085–14.834)		0.037	
1.188 (0.442–3.194)				0.73		0.596 (0.297–1.195)				0.15		0.379 (0.074–1.946)		0.25	
3.534 (1.279–9.055)				0.009		0.759 (0.386–1.490)				0.42		1.282 (0.289–5.676)		0.74	
1.009 (0.953–1.068)				0.76		0.986 (0.954–1.020)				0.42		0.857 (0.782–0.938)		0.001	

HR; hazard ratio, 95% CI; 95% confidence interval, VT; ventricular tachycardia, LVOT; left ventricular outflow tract, LGE; Late gadolinium enhancement

The composite clinical outcomes included all cause death, cardiac transplantation, cerebral stroke, heart failure requiring hospitalization including new onset atrial fibrillation, and defibrillator implantation.

### Additive predictive values of various prognostic models

To establish models for the prediction of composite clinical outcomes, variables that were either indicated to be significant by multivariable analysis (p < 0.20) or deemed clinically meaningful were selected and analyzed. MODEL 1 was composed of conventional factors, such as clinical variables and non-sustained VT on Holter monitoring. Selective parameters were added in a stepwise manner to MODEL 1 and the respective models were established. MODEL 2 included echocardiographic parameters (LVEDD, LAVI, and E/e`) on top of MODEL 1. A separate model (MODEL 2–1) where CMR parameters (the presence of LGE [> 15%LV] and LV ejection fraction on CMR) were added to MODEL 1 was set up and evaluated. MODEL 3 included the CMR parameters in addition to MODEL 2. MODEL 4 included CPET parameters (abnormal hemodynamic response to exercise and lower exercise capacity) added to MODEL 3.

**Fig 1:**
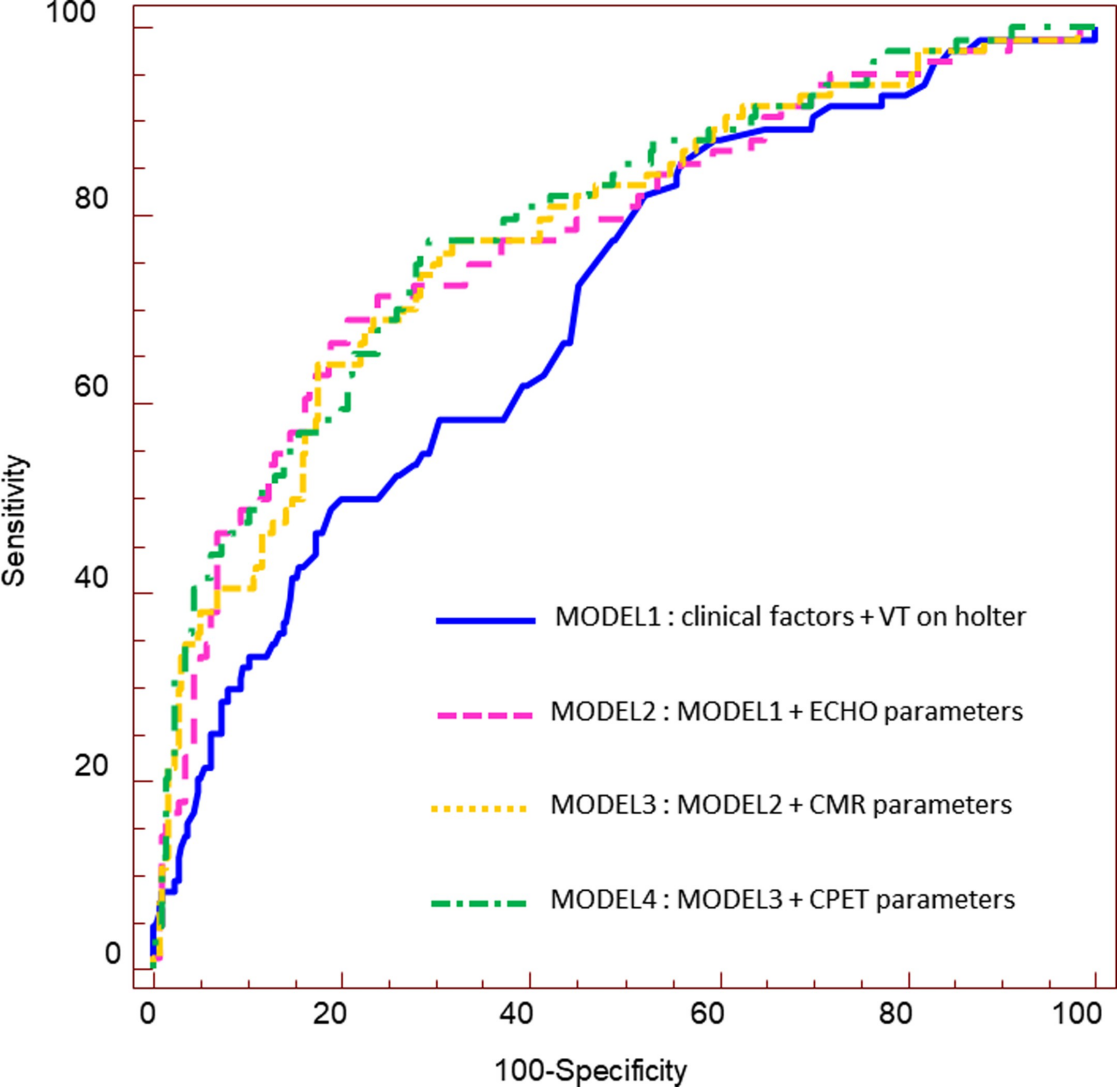
The comparison of ROC curve analysis. The reference model (MODEL 1) included age, sex, history of syncope as combined clinical parameters, and non-sustained ventricular tachycardia on Holter. Echocardiographic parameters were added to the left ventricular end diastolic dimension, left atrium volume index, and E/e’ ratio. Cardiac magnetic resonance parameters were included in addition to the presence of LGE (> 15%LV) and left ventricular ejection fraction. Cardiopulmonary exercise test parameters were included in addition to abnormal hemodynamic response to exercise and lower exercise capacity (peak VO_2_ ≤27.4 mL/Kg/min). Incremental prognostic value by ROC curve analysis was compared among 4 models. LGE: late gadolinium enhancement; LV: left ventricular; ROC: receiver operating characteristic curve.

The incremental prognostic value of non-sustained VT on Holter, echocardiographic parameters, CMT parameters, and CPET parameters over conventional clinical variables ranged from 0.700 to 0.790 on the basis of the value of the AUC obtained by the ROC curve analysis (Table 4 and Fig 1). All models showed statistical significance for the prediction of composite clinical outcomes as the NRI value (all p-values < 0.05) (Table 4) by risk reclassification analysis. Particularly, in Model 4, where CPET variables were added to clinical, echocardiographic, and CMR parameters, definite incremental values for composite outcome prediction were significantly identified (p-values < 0.005 on NRI and IDI values).

**Table 4:** Comparison of models with stepwise additive parameters in addition to combined clinical parameters.

	AUC	SE	95% CI	NRI (Category-free)	p-value	IDI (%)	p-value
MODEL1 (Reference model)	0.700	0.032	0.638–0.763				
age	0.607	0.034	0.540–0.673				
sex	0.517	0.036	0.446–0.588				
history of syncope	0.545	0.037	0.472–0.617				
history of non-sustained VT on holter	0.605	0.038	0.531–0.679				
MODEL2 (MODEL1 + ECHO parameters)	0.775	0.031	0.715–0.835	0.5538 (0.3180–0.7896)	< 0.001	9.40	< 0.001
MODEL2-1 (MODEL1 + CMR parameters)	0.714	0.032	0.650–0.777	0.4708 (0.2334–0.7081)	< 0.001	3.86	0.001
MODEL3 (MODEL2 + CMR parameters)	0.778	0.030	0.719–0.836	0.3578 (0.1178–0.5978)	0.003	1.61	0.064
MODEL4 (MODEL3 + CPET parameters)	0.790	0.029	0.733–0.846	0.3977 (0.1600–0.6353)	0.001	1.84	0.031

MODEL1 included age, sex, history of syncope, history of non-sustained VT on holter

ECHO parameters: LVEDD + LAVI + E/e’ ratio

CMR parameters: The presence of LGE (>15%LV) + LV EF on CMR

CPET parameters: Abnormal hemodynamic response to exercise + lower exercise capacity (peak VO2 ≤27.4 mL/Kg/min)

The variables for the multivariable model were selected, which were indicated variables from multivariable analysis with p<0.2 or clinically meaningful parameters. It was performed on the incremental prognostic value for the prediction of clinical outcomes by Net reclassification improvement (NRI) and integrated discrimination index (IDI).

95% CI; 95% confidence interval, AUC; area under the curve, SE; standard errors, NRI; net reclassification improvement, IDI; integrated discrimination index, VT; ventricular tachycardia, ECHO; echocardiography, LVEDD; Left ventricle end-diastolic dimension, LAVI; Left atrium volume index, CMR; cardiac magnetic resonance, LGE; Late gadolinium enhancement, LV EF; left ventricular ejection fraction, CPET; Cardiopulmonary exercise test

## Discussion

We investigated the relationship between conventional clinical variables and additional parameters obtained from multiple modalities and composite clinical outcomes during a mid-range follow-up period. Our data showed that in addition to the clinical and echocardiographic parameters, the extent of myocardial fibrosis and information on exercise capacity obtained from CPET could be useful additional significant predictors of composite clinical outcomes in patients with HCM.

In previous studies, several factors, such as LGE on CMR, LVOT obstruction, biomarkers, and diastolic dysfunction, may have contributed to the adverse clinical outcomes with reduced exercise capacity in patients with HCM [13–15, 24]. The reduced exercise capacity could be explained by the physiology of myocardial fibrosis and hemodynamic changes in myocardial stiffness.

Among them, LGE on CMR has been found to be associated with overall mortality [11, 13, 17, 37, 38] and malignant arrhythmias in patients with HCM [12]. Our findings confirmed that the presence and amount of myocardial fibrosis could be a determinant of composite clinical outcomes in patients with HCM, and could also add value to conventional factors. The uniqueness of our study was that we demonstrated that CPET may also be an important tool for risk stratification in HCM on top of CMR findings. Moreover, we showed that functional capacity demonstrated by peak VO_2_ was an independent predictor of outcomes in patients with HCM, with incremental value as a risk factor when added to the alleged parameters.

Patients with HCM frequently exhibit limited exercise capacity [39–41]. The reduced exercise capacity may be attributed to the inability to increase stroke volume. This inability is due to impaired diastolic filling and LV contractile reserve for overcoming afterload [25, 42]. Exercise stress testing has previously been used to assess clinical risk [31, 43]. Here the most well-accepted prognostic factor for HCM in exercise physiology is exercise-induced abnormal BP response. This abnormal hemodynamic response can be attributed to marked LV systolic dysfunction, decreased cardiac output, and hypotension [44]. However, most studies on exercise physiology in HCM have focused on hemodynamic responses and simple exercise duration. We described the role of advanced modalities in obtaining additional risk stratification for future clinical events using all elements combined.

Patients with HCM have a high risk of sudden cardiac death; therefore, the guidelines suggest major clinical features associated with an increased risk of sudden cardiac death in adults [18]. However, in our results, there were not many events of cardiac death or sudden cardiac death because of registry data. Among the composite outcomes, heart failure events accounted for the highest rate. Therefore, our results suggest that for predicting and managing life quality and morbidity, the predictive value of functional changes, such as exercise capacity, was more statistically significant than that of pathologic changes such as fibrosis, which has been found to be associated with cardiac death. According to the results of previous studies, many methods have been suggested to increase the survival rate of patients with HCM. However, as for patients who are older or ageing, heart failure-related events such as repeated admission may be as important as sudden death, as elderly HCM patients such as subjects in our group are not prone to exhibit high rates of sudden cardiac death as their younger counterparts according to previous reports [45, 46]. As this is the case, our results may be especially important in managing older HCM patients who are increasing in number as the longevity of the general population is increasing.

Patients with HCM can present with a wide and varied spectrum of symptoms, including exertional shortness of breath, chest pain, syncope, or pre-syncope. In many situations, symptoms are equivocal. Cardiopulmonary exercise stress testing provides not only customized objective information on exercise capacity, but also information related to exertional symptoms that appear during daily activities. This is notable, as exercise-related subjective symptoms, which can be vague or equivocal, are key factors of concern during treatment planning. Objective estimation of exercise capacity and symptoms will provide a clearer picture of the patient’s clinical status. Exercise stress testing in patients with HCM is safe and rarely leads to serious adverse events [47, 48]. Therefore, we emphasize that cardiopulmonary exercise stress testing would be highly useful for patients with HCM. As our study showed, exercise capacity and hemodynamic response on CPET have additive values for predicting clinical outcomes. Performing CPET in patients with HCM would be helpful in determining both clinical outcomes and treatment plans.

### Limitations

The first limitation of this study is that it was a single-center observational study. However, being conducted at a tertiary hospital, follow-up was performed on the entire study population meticulously so that we could detail the outcomes and associate them closely with baseline characteristics. Second, we included only patients who were able to perform exercise testing, which may have been the reason for the relatively small number of cases of cardiac death (n = 3) and all-cause death (n = 9). However, as patients with HCM are generally mildly symptomatic, and risk stratification for HCM was performed in relatively active patients in general, we considered our results to be acceptable for generalization in the real world. Third, we did not include additional data on post-exercise hemodynamic parameters, such as chronotropic incompetence or exercise-induced arrhythmia in our study [49]. Instead, we focused on the most widely used simple parameters for estimating exercise capacity objectively and tried not to overcomplicate the already complicated method of risk stratification in HCM.

## Conclusions

This study demonstrated that CPET-derived peak oxygen consumption level, especially when combined with LGE on CMR, added value to conventional clinical risk factors as prognostic factors in patients with HCM, especially in terms of heart failure-related admissions. Dynamic objective assessment of patients with CPET may aid in elucidating various symptoms, improving treatment planning, and assessing risk stratification in patients with HCM.
